# Multiscale Characterization of Rice Starch Gelation and Retrogradation Modified by Soybean Residue (Okara) and Extracted Dietary Fiber Using Rheology, Synchrotron Wide-Angle X-Ray Scattering (WAXS), and Fourier Transform Infrared (FTIR) Spectroscopy

**DOI:** 10.3390/foods14111862

**Published:** 2025-05-23

**Authors:** Aunchalee Aussanasuwannakul, Suparat Singkammo

**Affiliations:** 1 Department of Food Chemistry and Physics, Institute of Food Research and Product Development, Kasetsart University, Bangkok 10903, Thailand; 2 Synchrotron Light Research Institute (Public Organization), 111 University Avenue, Muang District, Nakorn Ratchasima 30000, Thailand; suparat@slri.or.th

**Keywords:** okara, dietary fiber, rice starch, retrogradation, rheology, WAXS, FTIR, starch gelation, gluten-free formulation, food waste valorization

## Abstract

Okara, the soybean residue from soy milk and tofu production, offers significant potential as a sustainable, fiber-rich ingredient for starch-based and gluten-free food systems. This study investigates the comparative effects of whole okara and its extracted dietary fiber (DF) on the retrogradation, rheological properties, and nanostructural organization of rice starch (RS) gels. Rice starch suspensions were blended with 5–20% (dry basis) of either whole okara or DF, thermally gelatinized, and analyzed using dynamic rheology, synchrotron-based Wide-Angle X-ray Scattering (WAXS), and Fourier Transform Infrared (FTIR) spectroscopy. DF markedly reduced the gelation temperature and enhanced storage modulus (G′), indicating earlier and stronger gel network formation. WAXS analysis showed that DF more effectively disrupted long-range molecular ordering, as evidenced by suppressed crystallinity development and disrupted molecular ordering within the A-type lattice. FTIR spectra revealed intensified O–H stretching and new ester carbonyl bands, with progressively higher short-range molecular order (R_1047/1022_) in DF-modified gels. While whole okara provided moderate retrogradation resistance and contributed to network cohesiveness via its matrix of fiber, protein, and lipid, DF exhibited superior retrogradation inhibition and gel stiffness due to its purity and stronger fiber–starch interactions. These results highlight the functional divergence of okara-derived ingredients and support their targeted use in formulating stable, fiber-enriched, starch-based foods.

## 1. Introduction

The rising consumer demand for gluten-free and plant-based foods has driven interest in novel functional ingredients that meet both dietary and sustainability objectives [1,2]. Rice starch is particularly attractive in gluten-free applications due to its clean flavor, hypoallergenic nature, and favorable gelatinization properties [3,4]. However, its high retrogradation tendency—characterized by the recrystallization of amylose and amylopectin during storage—remains a major limitation, leading to increased firmness, reduced water retention, and shortened shelf life [5,6,7,8]. While retrogradation is widely studied, its structural mechanisms remain insufficiently understood in fiber-enriched starch systems, where molecular interactions are more complex. Similar retrogradation challenges have been reported in starch-based gels from wheat, corn, and potato, where both short- and long-range structural changes were found to impact texture and stability during storage [9,10]. This study applies a multiscale analytical approach—including dynamic rheology, synchrotron-based Wide-Angle X-ray Scattering (WAXS), and Fourier transform infrared (FTIR) spectroscopy—to examine how different fiber types influence starch gelation and structural reorganization at macro-, micro-, and nanoscale levels.

Dietary fibers have emerged as promising modulators of starch retrogradation. In rice starch systems, fibers can alter gelatinization behavior, modulate water mobility, and form hydrogen bonds with starch chains, thereby suppressing recrystallization and stabilizing gel structure [11,12]. Recent studies demonstrate that soluble and insoluble fibers can suppress retrogradation through mechanisms such as water immobilization, steric hindrance, and disruption of chain realignment [13,14,15,16]. Moreover, researchers have shown that hemicellulose, cellulose, and lignin differentially affect the recrystallization of amylopectin, with hemicellulose being most effective in delaying retrogradation [17,18]. However, fiber effects depend on composition, processing history, and interaction with starch components, and few studies have systematically compared the effects of complex fiber matrices versus purified fiber fractions on starch retrogradation [19,20,21].

Okara, the fiber- and protein-rich byproduct of soy milk and tofu production, presents an upcycled ingredient opportunity aligned with circular economy goals [22,23,24,25]. Composed of 40–60% dietary fiber and 20–30% protein, okara offers both nutritional and functional value but remains underutilized. Advances in fractionation techniques have enabled the isolation of okara’s dietary fiber (DF) fraction, which may exhibit distinct structural functionality compared to the whole matrix. While prior work has demonstrated the use of okara and DF in food matrices, only Zhuang et al. (2024) [26] have directly compared their effects in starch gels—specifically potato starch—highlighting a research gap for rice starch, which differs markedly in granule morphology, crystallinity, and amylopectin architecture [15,16,27,28].

Given these differences, understanding the distinct functional roles of whole okara and its DF in rice starch systems is essential. Industry adoption of okara-derived ingredients hinges on demonstrating their ability to modulate retrogradation, stabilize gel structure, and support shelf-life extension. However, comparative studies exploring both macro-level performance (e.g., rheology) and micro-to-nanoscale structure (e.g., WAXS, FTIR) remain scarce. This study addresses this gap by systematically comparing how whole okara and its extracted DF modulate gelation, retrogradation, and nanostructural features of rice starch gels across a range of substitution levels.

To elucidate these interactions, synchrotron WAXS was used to monitor crystallite development and molecular ordering during and after thermal gelation. This high-resolution method captures changes in crystallinity, crystallite size, diffraction symmetry, and peak broadening that are not detectable via conventional XRD [29]. Complementing WAXS, FTIR spectroscopy was employed to probe molecular-level changes—particularly hydrogen bonding dynamics and ester carbonyl incorporation within the starch matrix—features that can be reliably detected using FTIR [30] that accompany fiber–starch interactions. Together with dynamic rheological testing, this multiscale toolkit captures how okara and DF influence structure–function relationships in starch gels.

The central hypothesis guiding this study is that whole okara and extracted DF exert distinct effects on the retrogradation and gelation of rice starch due to their compositional and physicochemical differences. Whole okara, which contains proteins, lipids, and insoluble fiber, may contribute to network cohesiveness and moisture retention, while DF, rich in polysaccharide chains and devoid of interfering non-fiber components, may serve as a more efficient inhibitor of starch retrogradation through enhanced hydrogen bonding and matrix densification. Accordingly, this study investigates the following points: (1) how okara and DF affect gelation temperature, viscoelastic moduli, and network stability; (2) how they alter crystalline structure and ordering, as revealed by synchrotron WAXS; and (3) how they modify molecular interactions within the starch matrix, as detected by FTIR. The findings are intended to support the design of fiber-enriched, clean-label starch systems and advance the functional valorization of okara in sustainable food applications.

## 2. Materials and Methods

### 2.1. Materials and Sample Preparation

**Figure 1:**
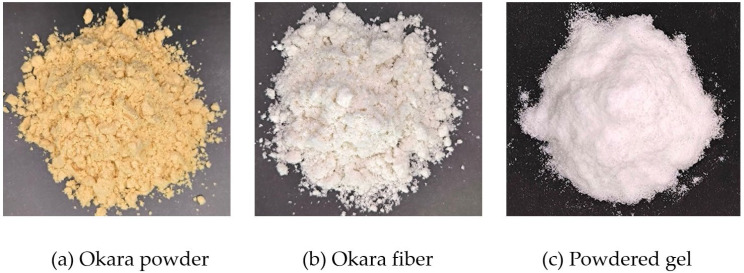
Visual appearance of materials used in this study: (**a**) dried whole okara powder used in RSO samples; (**b**) extracted dietary fiber from okara used in RSF samples; and (**c**) freeze-dried powdered gel (example: RSF-10) used for WAXS and FTIR analyses.

Rice starch was obtained from a commercial supplier (Cho Heng Rice Vermicelli Factory Co., Ltd., Nakhon Pathom, Thailand), with an amylose content of at least 23%. Fresh okara was sourced from a local tofu/soymilk manufacturer (NGOWJENGNGOUN Co., Ltd., Bangkok, Thailand), then tray-dried at 60 °C until the moisture content fell below 10%, ground into powder, and sieved through a 100-mesh sieve. The resulting okara powder used in this study contained 4.33% moisture, 29.35% protein, 12.85% fat, 1.28% soluble dietary fiber (SDF), and 36.93% insoluble dietary fiber (IDF), corresponding to a total dietary fiber content of 38.21% on an as-is basis (Figure 1a).

Dietary fiber (DF) was extracted from okara by suspending 20.0 g of okara powder in 100 mL of 5% NaOH solution and incubating at 80 °C for 1 h. This alkali treatment was selected based on its proven efficiency in extracting hemicellulose I-type polysaccharides with high insoluble fiber content and low protein contamination, suitable for studying structural effects in starch-based systems and use in rheological, WAXS, and FTIR analyses, as detailed in Section 2.2, Section 2.3 and Section 2.4. After cooling to 45–50 °C, the pH was adjusted to 8.0, followed by enzymatic treatment with 0.3% (*w*/*v*) trypsin (porcine pancreas; Tokyo Chemical Industry Co., Ltd., Tokyo, Japan) at 45 °C for 3 h. The enzyme was inactivated at 90 °C for 10 min. The residue was recovered by centrifugation, washed thoroughly, freeze-dried, and ground into fine powder (Figure 1b). The extraction yield was approximately 28.3% (dry basis). The extracted DF powder used in this study contained 2.58% moisture, 0.87% protein, 6.67% fat, 3.15% ash, 82.89% insoluble dietary fiber, and 2.58% soluble dietary fiber (dry basis). Chelating agents, such as sodium citrate, which are suitable for selective extraction of pectic polysaccharides, were not used in this study, as the aim was to obtain a broad-spectrum dietary fiber fraction with structural functionality rather than to isolate specific soluble fiber components.

Rice starch was blended with either whole okara or extracted dietary fiber (DF) at substitution levels of 0%, 5%, 10%, 15%, and 20% (*w*/*w* on a dry starch basis). These blends are hereafter referred to as RS (100% rice starch), RSO-5 to RSO-20 (5–20% okara substitution), and RSF-5 to RSF-20 (5–20% dietary fiber substitution), respectively.

### 2.2. Rheological Analyses

For rheological measurements, higher concentration suspensions were required to produce stronger gels suitable for oscillatory testing. Therefore, samples were prepared at 25% (*w*/*v*), as described by Liu et al. (2016) [31]. For the control (0% addition), 500 mg of rice starch was mixed with 2.0 mL of distilled water. For fiber-substituted formulations, 475 mg of rice starch was combined with 25 mg of okara or DF and suspended in 2.0 mL of water to maintain the same total solids content. This higher concentration enabled robust viscoelastic responses during temperature and frequency sweep tests by minimizing flow artifacts and ensuring that measurements were conducted within the linear viscoelastic region, as verified through preliminary strain sweep testing.

Dynamic oscillatory rheological properties were measured using a modular compact rheometer (MCR 302, Anton Paar, Graz, Austria) equipped with parallel-plate geometry (25 mm diameter, 1 mm gap). All measurements followed the protocol of Aussanasuwannakul et al. (2024) [32], with modifications. Each sample suspension was vortexed for 5 min before being loaded onto the rheometer. After loading, the sample was conditioned at 25 °C for 1 min prior to testing. Two rheological tests were conducted following the methodology of Liu et al. (2016) [31].

#### 2.2.1. Temperature Sweep

**Figure 2:**
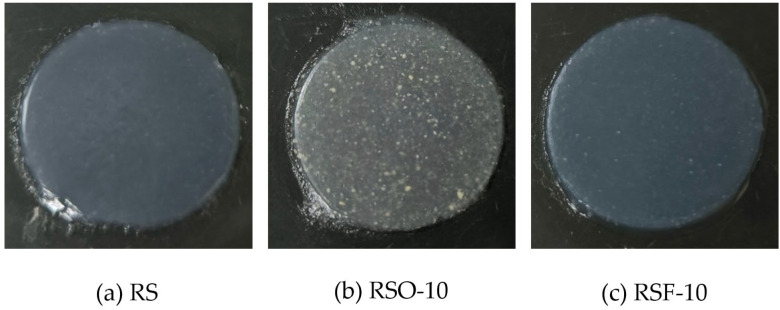
Examples of gelatinized rice starch mixtures used for rheological measurements: (**a**) RS (100% rice starch); (**b**) RSO-10 (10% okara substitution); (**c**) RSF-10 (10% dietary fiber substitution).

The test was conducted at a constant strain of 2% and an angular frequency of 10 rad/s. After equilibration at 25 °C for 1 min inside the rheometer’s Peltier-controlled measuring system, the temperature was ramped up from 25 to 95 °C and then cooled back to 25 °C at a rate of 5 °C/min. The resulting gel (Figure 2) was equilibrated at 25 °C before frequency sweep analysis. During the temperature sweep, the storage modulus (G′) and loss modulus (G″) were recorded continuously as a function of temperature. The gelation (crossover) temperature (Tc) was identified from the heating phase, where G′ = G″.

#### 2.2.2. Frequency Sweep

Measurements were performed at 25 °C over an angular frequency range of 0.1 to 100 rad/s, using a strain of 2% within the linear viscoelastic (LVE) region. Three measuring points per decade were used. Storage modulus (G′) and loss modulus (G″) were recorded as functions of angular frequency. Frequency dependence (slopes of log G′ vs. log ω), complex modulus (G*), and tan δ (G″/G′) were calculated to assess the viscoelastic behavior and structural stability of the gels.

All data were collected and analyzed using RheoCompass™ software (Version 1.30, Anton Paar GmbH, Graz, Austria). All rheological measurements were performed in triplicate.

### 2.3. Wide-Angle X-Ray Scattering (WAXS) Analysis

For structural characterization using Wide-Angle X-ray Scattering (WAXS) and Fourier transform infrared (FTIR) spectroscopy, 8% (*w*/*v*) suspensions were prepared by dispersing 4.0 g of each starch–fiber mixture in 50 mL of distilled water, following the method of Zhuang et al. (2024) [26]. This concentration facilitated uniform gelatinization and freeze-drying. Heated samples were cooled, freeze-dried, and ground into a fine powder for analysis (Figure 1c).

**Figure 3:**
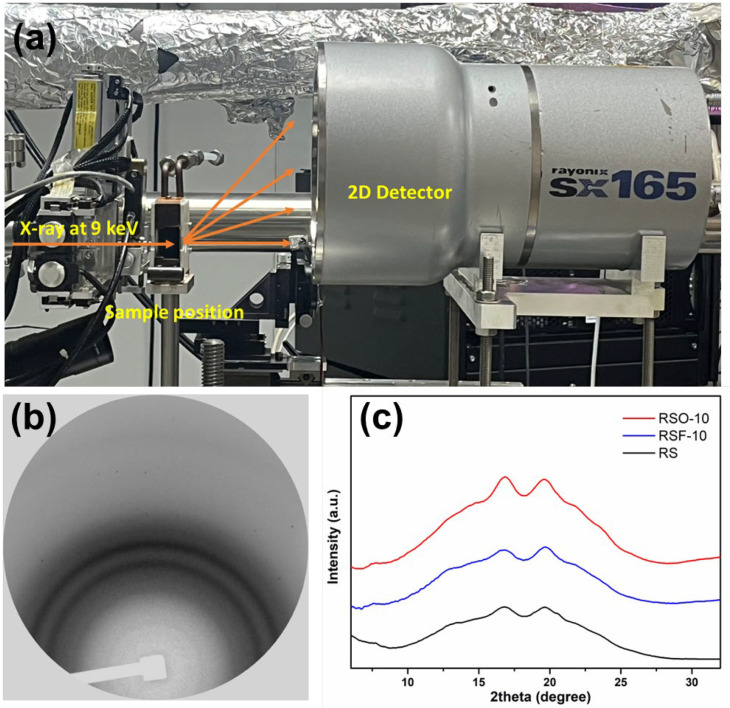
WAXS experimental setup and representative results: (**a**) schematic of the WAXS experimental setup at BL1.3W, SLRI, Thailand; (**b**) two-dimensional scattering pattern of RSF-10; (**c**) overlay of 1D WAXS intensity profiles for RS, RSO-10, and RSF-10, highlighting the impact of okara and dietary fiber addition on diffraction intensity and peak characteristics.

Wide-Angle X-ray Scattering (WAXS) measurements were carried out at the SAXS beamline (BL1.3W) of the Synchrotron Light Research Institute (SLRI), Thailand (Figure 3a). Finely ground samples were uniformly loaded into an aluminum sample holder and sealed with Kapton films to minimize moisture loss during measurement. The samples were irradiated with monochromatic X-rays of wavelength 0.137 nm (corresponding to an energy of 9 keV), with a sample-to-detector distance of 210 mm and an exposure time of 600 s at an ambient temperature. The scattered X-rays were collected using a Rayonix SX165 CCD detector. Raw two-dimensional (2D) scattering patterns (Figure 3b) were preprocessed using SAXSIT software (version 4.63) to extract one-dimensional (1D) intensity profiles (Intensity vs. q). To facilitate comparison with conventional X-ray diffraction (XRD) studies, the data were converted from scattering vector q (Å^−1^) to 2θ angles (°) based on the Cu Kα equivalent wavelength (λ = 0.154 nm) (Figure 3c).

The processed 1D WAXS profiles were analyzed to investigate the effect of whole okara and its extracted dietary fiber (DF) on the crystalline structure and retrogradation behavior of rice starch gels. Multiple structural parameters were extracted from the WAXS profiles. The crystallinity percentage was determined by fitting the diffraction peaks using a pseudo-Voigt function to separate the crystalline contribution from the amorphous background.

### 2.4. Fourier Transform Infrared (FTIR) Spectroscopy

Fourier transform infrared (FTIR) spectroscopy was used to investigate the molecular interactions and structural organization of rice starch (RS) gels incorporated with whole okara (RSO) and extracted dietary fiber (RSF). Spectra were obtained using an ATR-FTIR (Attenuated Total Reflectance Fourier transform infrared) spectrometer (Vertex 70, Bruker, Billerica, MA, USA) operating in the range of 4000–400 cm^−1^. Each sample was scanned 32 times at a resolution of 4 cm^−1^.

The gel samples were first freeze-dried and finely ground. Approximately 2–3 mg of sample was gently compressed onto the diamond crystal of the ATR module using a constant-pressure clamp to ensure consistent sample–crystal contact across all measurements. No additional chemical preparation or dilution was applied. Spectra were baseline corrected and vector normalized before analysis to reduce variability.

Key absorption bands—including O–H stretching (~3280–3296 cm^−1^), C–H stretching (~2925 cm^−1^), and ester carbonyl C=O stretching (~1745 cm^−1^)—were examined to assess hydrogen bonding and functional group incorporation. The R_1047/1022_ ratio, derived from the fingerprint region (1200–900 cm^−1^), was calculated to evaluate short-range molecular order in the starch matrix, following the approach described by Zhuang et al. (2024) [26]. Given the nature of ATR-FTIR, this ratio is interpreted as a relative indicator of molecular ordering rather than an absolute metric.

### 2.5. Statistical Analysis

Dynamic rheological measurements were conducted in triplicate for each sample. The Tc and **G′** at 0.01, 1, and 100 rad/s are reported as mean ± standard deviation (SD). To evaluate differences in gelation temperature and viscoelastic parameters across treatments, one-way analysis of variance (ANOVA) followed by Tukey’s Honestly Significant Difference (HSD) test was applied, with significance defined at *p* < 0.05.

For synchrotron WAXS and FTIR analyses, due to beamline access limitations and instrument throughput constraints, each treatment was analyzed in two independent replicates. The crystallinity (%) and R_1047/1022_ ratios are reported as average values of these replicates. The coefficient of variation (CV) remained below 10%, indicating high consistency between measurements. However, no inferential statistics were applied to WAXS and FTIR data due to the limited sample size. Accordingly, the interpretation of structural and molecular trends in these analyses remains exploratory and descriptive, focusing on consistent profile changes across treatments rather than on formal statistical differentiation.

## 3. Results

### 3.1. Dynamic Rheological Properties

**Figure 4:**
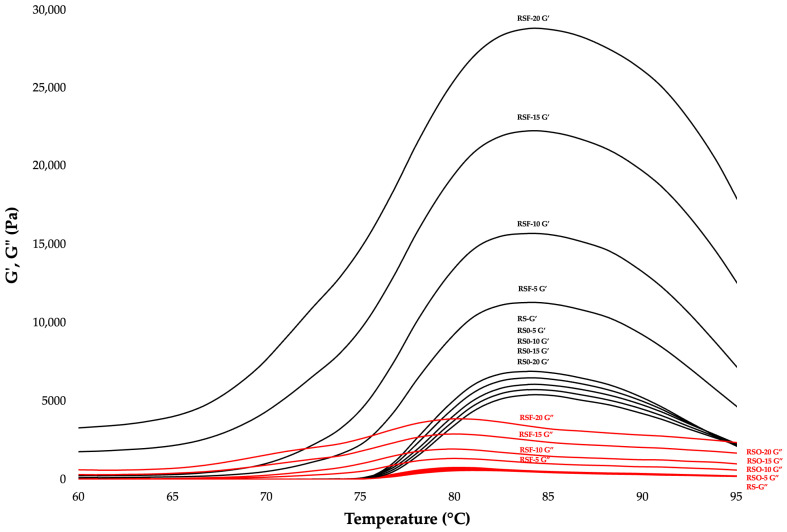
Temperature sweep profiles of rice starch (RS), RS–okara (RSO), and RS–dietary-fiber (RSF) gels. Storage modulus (G′) and loss modulus (G″) plotted as a function of temperature. Arrows indicate gelation temperature (Tc), where G′ = G″. Incorporation of okara and DF led to significant reductions in Tc, suggesting accelerated gelation.

The dynamic rheological measurements provided insights into the gelation dynamics and structural integrity of rice starch (RS) gels containing whole okara (RSO) and extracted dietary fiber (RSF). The temperature sweep analysis showed that the gelation temperature (Tc), defined as the crossover point of storage (G′) and loss (G″) moduli, was 75.3 ± 0.12 °C for pure RS. The incorporation of okara and RSF significantly reduced Tc, decreasing to approximately 66.7 °C in the RSO series (RSO-10, RSO-15, and RSO-20) and further down to 59.6 °C in the RSF series (RSF-5 to RSF-20) (Figure 4; Table 1). This reduction in Tc suggests that both whole okara and RSF facilitated earlier gel network formation, potentially by promoting phase separation and enhancing amylose interactions during thermal transition.

To evaluate the mechanical integrity of the gel network, frequency sweep tests were conducted from 0.1 to 100 rad/s. Across all samples, G′ consistently exceeded G″, indicating solid-like behavior and the formation of structured gel networks. The log–log plots of G′ and G″ versus angular frequency were nearly parallel and showed minimal slope, characteristic of robust viscoelastic gels with high resistance to mechanical deformation. At a low frequency (0.01 rad/s), G′ values surpassed 10 Pa in all treatments, confirming the development of stable three-dimensional networks.

**Figure 5:**
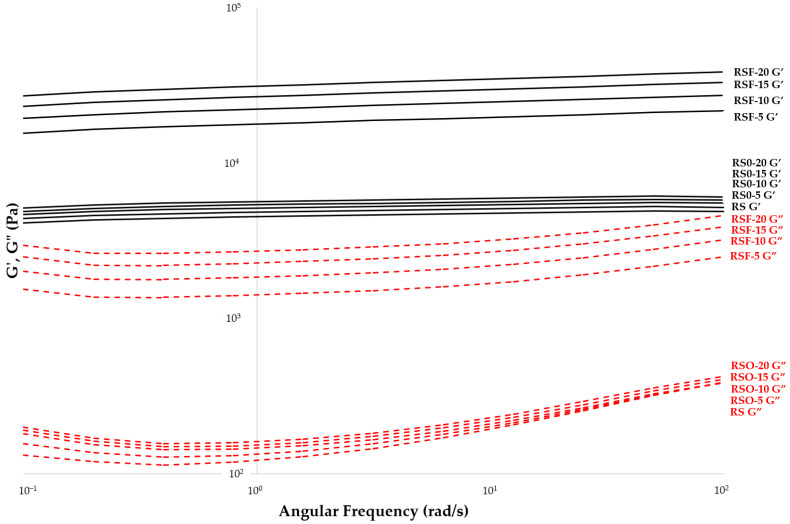
Frequency sweep profiles of rice starch (RS), RS–okara (RSO), and RS–dietary-fiber (RSF) gels. G′ and G″ plotted across an angular frequency range of 0.1–100 rad/s at 25 °C. All samples demonstrated stable gel-like behavior (G′ > G″). RSF gels showed significantly higher G′ values, indicating stronger network structures.

At an angular frequency of ~1 rad/s, representative of mechanical stress encountered in many food applications, the G′ values increased from 4480 Pa in RS to 5595 Pa in RSO-20, and up to 30,700 Pa in RSF-20 (Figure 5; Table 1). This substantial enhancement in G′ for RSF-treated samples demonstrates the superior gel-strengthening capacity of purified dietary fiber compared to whole okara. The frequency dependence of G′ (i.e., the slope of log G′ vs. log ω) remained low (<0.05) in all samples, supporting the formation of frequency-independent gel structures with stable elastic properties.

Moreover, the dominance of G′ over G″ and the low values of tan δ across the frequency spectrum indicate that all formulations retained an elastic-dominant behavior. Such rheological profiles are favorable for food systems requiring textural integrity and long-term stability. These observations suggest that RSF contributes not only to early network formation, but also to enhanced rigidity and resistance to structural breakdown. In contrast, whole okara may provide softer, more cohesive gels due to the presence of proteins and lipids, which interact with the starch matrix in distinct ways.

### 3.2. WAXS Results and Crystallinity Trends

Wide-Angle X-ray Scattering (WAXS) was employed to qualitatively examine the long-range molecular ordering of rice starch (RS) and its binary mixtures with whole okara (RSO) and extracted dietary fiber (RSF). All samples retained characteristic A-type diffraction peaks centered near 16–17° 2θ (Cu Kα), indicating that the primary starch crystalline polymorph remained after fiber incorporation. It should be noted that the A-type pattern corresponds to a monoclinic unit cell structure with B2 symmetry, typical of cereal starches such as rice [34,35]. This structural framework serves as the basis for interpreting changes in molecular ordering and crystalline domain disruption upon fiber addition.

**Figure 6:**
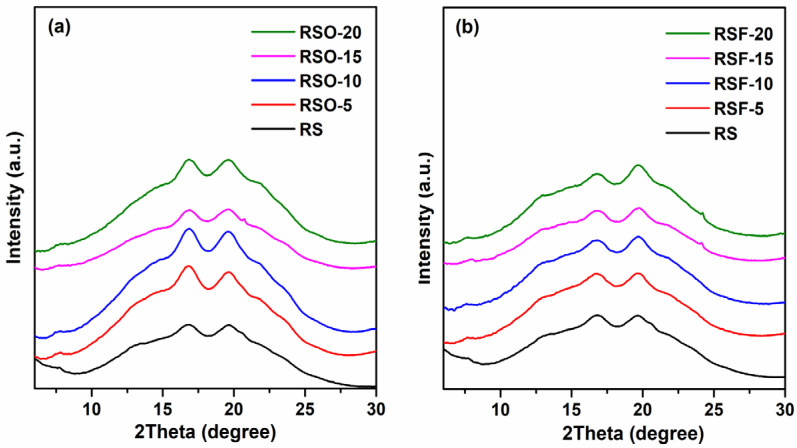
Overlay of 1D WAXS patterns for (**a**) rice starch (RS) and rice starch with different levels of okara (RSO-5, RSO-10, RSO-15, RSO-20), and (**b**) RS with different levels of extracted dietary fiber (RSF-5, RSF-10, RSF-15, RSF-20). Angle positions were converted to Cu Kα wavelength for comparison. Original 1D diffraction profiles in both 2θ and q space are provided in Appendix A (Figure A1) for data transparency and qualitative comparison.

As shown in Figure 6, the RSF and RSO samples exhibited broader, lower-intensity diffraction profiles compared to native RS. These visual differences suggest a decrease in crystalline domain perfection and reduced molecular ordering, particularly in RSF samples, which may reflect greater steric hindrance or disruption of starch double helices.

**Figure 7:**
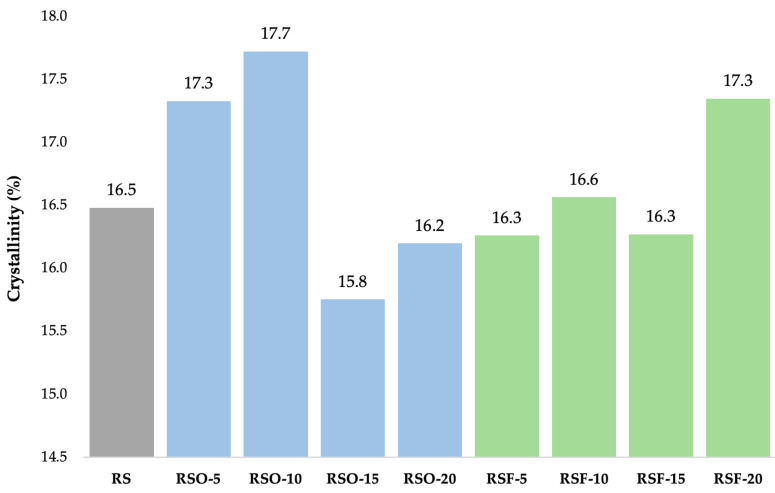
Crystallinity (%) derived from synchrotron WAXS profiles of rice starch (RS), okara-incorporated (RSO), and dietary-fiber-incorporated (RSF) gel samples. Fiber addition modulated crystallinity in a concentration-dependent manner, with RSF-20 showing the highest ordering. Values represent means from two independent replicates (CV < 10%). Results are presented as comparative trends rather than statistically tested differences.

As shown in Figure 7, fiber addition influenced crystallinity percentages across treatments. Crystallinity slightly increased at low okara levels (RSO-5 and RSO-10), then decreased at higher levels (RSO-15), while RSF samples generally maintained or slightly enhanced crystallinity, peaking at 17.35% in RSF-20. These patterns suggest altered molecular packing and localized ordering due to fiber interactions.

In the RSF series, the crystallinity values remained relatively stable across RSF-5 (16.26%), RSF-10 (16.56%), and RSF-15 (16.27%), with a slight increase at RSF-20 (17.35%). This suggests that the extracted dietary fiber did not markedly alter the degree of crystallinity at lower concentrations but slightly promoted localized recrystallization at higher levels.

### 3.3. FTIR Analysis

**Figure 8:**
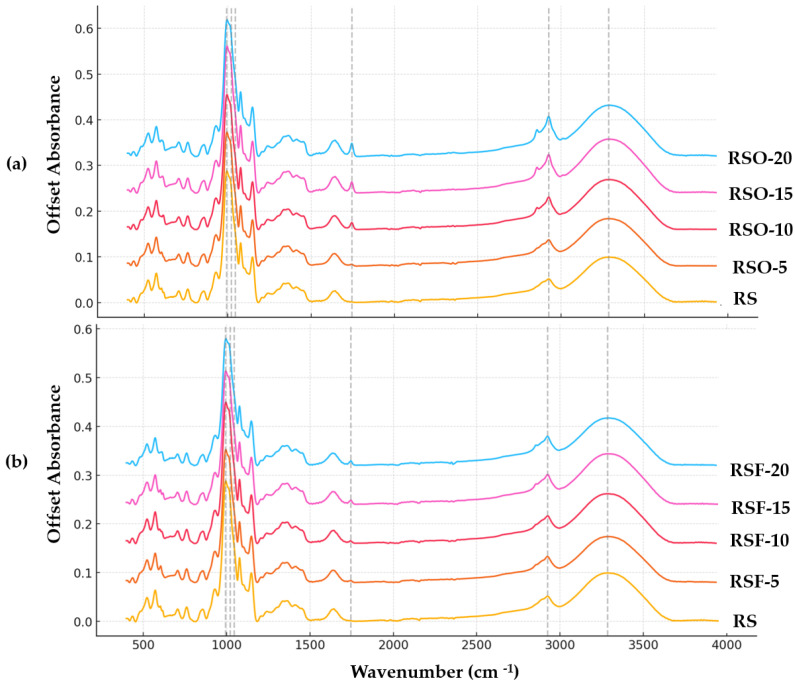
(**a**) FTIR spectra of rice starch gels incorporated with whole okara (RSO) at 5–20% substitution levels (*w*/*w*, dry basis). (**b**) FTIR spectra of rice starch gels incorporated with extracted dietary fiber (RSF) at 5–20% substitution levels (*w*/*w*, dry basis). Spectra are vertically offset for visual clarity; relative absorbance intensities remain unaltered. Sample names are labeled directly beside each spectrum. Enhanced O–H stretching (3280–3296 cm^−1^) and ester carbonyl (C=O) stretching (~1745 cm^−1^) were observed in modified samples. Differences in molecular interactions and structural ordering are evident in the fingerprint region (1200–900 cm^−1^). Horizontal reference lines assist visual comparison across treatments.

Fourier transform infrared (FTIR) spectroscopy was employed to investigate molecular-level changes in rice starch (RS) gels containing whole okara (RSO) and extracted dietary fiber (RSF) at 5–20% substitution levels (*w*/*w*, dry basis). Representative FTIR spectra are presented in Figure 8a (RS vs. RSO series) and Figure 8b (RS vs. RSF series). All samples exhibited a broad band in the 3280–3296 cm^−1^ range, assigned to O–H stretching vibrations, which reflects the extent of hydrogen bonding within the system. The band intensity increased upon fiber addition, particularly in RSF-5 and RSF-20, suggesting enhanced hydrogen bonding between starch chains and fiber components.

A distinct absorption band also appeared at ~1745 cm^−1^ in all RSO and RSF samples but was absent in the native RS control. This band is attributed to the C=O stretching vibration of ester carbonyl groups, likely originating from uronic acids or acetylated hemicellulose residues in the fiber fractions. Its consistent presence confirms the chemical contribution of okara-derived fiber materials to the starch matrix.

**Figure 9:**
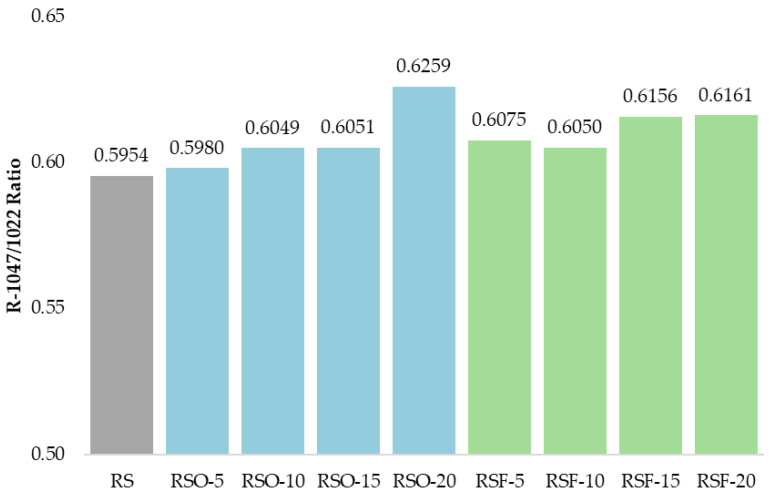
R_1047/1022_ ratios quantifying short-range molecular order in rice starch (RS), okara-incorporated (RSO), and dietary-fiber-incorporated (RSF) gel samples. The bar chart shows a general increase in structural order with increasing fiber substitution levels (5–20%, *w*/*w*, dry basis). RSF samples demonstrated higher ratios than RSO at equivalent levels, particularly at lower concentrations, suggesting a stronger enhancement of crystalline–amorphous phase balance by extracted dietary fiber. Values represent means from two independent replicates (CV < 10%). Results are presented as comparative trends rather than statistically tested differences.

In the fingerprint region (1200–900 cm^−1^), which encompasses the skeletal vibrations of polysaccharide backbones, the peak near 995 cm^−1^—often associated with the helical order of starch—remained visible across all treatments. Two key bands at 1047 cm^−1^ and 1022 cm^−1^ were selected to calculate the absorbance ratio R_1047/1022_, frequently used as an empirical indicator of short-range molecular order in starch systems. As summarized in Figure 9, this ratio increased across all fiber-containing samples relative to the RS control (0.5954), with the highest values observed in RSF-20 (0.6161) and RSO-20 (0.6259).

It should be noted, however, that the R_1047/1022_ ratio must be interpreted with caution. Although widely used in food and materials science to reflect the balance between crystalline and amorphous domains, this ratio does not arise from independent vibrational modes in a strict physical sense. The overlapping contributions from molecular conformations, baseline correction, and hydrogen bonding environments may affect these bands. Therefore, while R_1047/1022_ can provide insight into relative ordering trends, it should not be regarded as a direct quantitative measure of crystallinity. A critical perspective on this approach and its limitations is provided in the discussion (Section 4.3).

## 4. Discussion

This study aimed to elucidate how whole okara and its extracted dietary fiber (DF) differentially influence the retrogradation behavior, rheological properties, and nanostructural characteristics of rice starch (RS)-based gels. The findings support the primary hypothesis: whole okara and DF exert distinct functional effects due to differences in composition, water-binding capacity, and structural interactions with starch.

### 4.1. Gelation Behavior and Viscoelastic Properties

The rheological behavior of rice starch gels was significantly influenced by the incorporation of whole okara and extracted dietary fiber (DF), revealing compositional and structural modifications in gelation and viscoelasticity. The temperature sweep data indicated that the gelation temperature (Tc) of rice starch (RS) was 75.3 ± 0.12 °C, decreasing to approximately 66.7 °C in all RSO samples and further to 59.6 °C in RSF samples. This trend aligns with reports that fiber inclusion promotes early amylose aggregation by altering hydration dynamics and promoting phase separation [17,31]. In high-amylose starch systems (~23% amylose in this study), such early aggregation facilitates the formation of denser gel networks during cooling and storage [11].

Frequency sweep tests revealed significant differences in gel strength and elasticity between the treatments. Both okara and DF enhanced the storage modulus (G′) of the rice starch gels, with more pronounced effects being observed in the RSF samples. At an angular frequency of 1 rad/s, G′ increased from 4480 Pa in RS to 5595 Pa in RSO-20 and dramatically to 30,700 Pa in RSF-20. These results are consistent with those of recent studies, demonstrating that insoluble dietary fiber enhances starch gel strength by promoting interfacial adhesion, water sequestration, and structural reinforcement within the matrix [17,26,36]. Insoluble dietary fiber (IDF), which makes up 82.89% of the extracted DF powder used in this study, acts as a physical scaffold that entraps water and facilitates hydrogen bonding with leached starch chains, thereby reinforcing the elastic network [16,37].

The low frequency dependence (log G′ slope < 0.05) observed across all treatments confirmed the formation of stable, frequency-insensitive gel networks. Such behavior is typical of highly structured biopolymer gels and is supported by earlier studies on cellulose- and hemicellulose-enhanced starch systems, which report improved long-term stability and reduced syneresis [17,33]. Hemicellulose, although not explicitly quantified in this study, may be present in small amounts in the DF fraction and could contribute synergistically to the gel network’s strength.

Interestingly, the rheological enhancement by whole okara was modest compared to that of DF but showed a different quality of reinforcement. RSO samples exhibited increased cohesiveness and reduced brittleness, likely due to the complex composition of whole okara, which includes 29.35% protein and 12.85% fat in addition to fiber. Proteins may interact with starch through hydrogen bonding or steric hindrance, forming elastic bridges that enhance network integrity [38,39]. Meanwhile, the lipid fraction may participate in amylose–lipid complex formation, potentially disrupting the alignment of starch chains and reducing retrogradation-induced hardening [40]. These protein–lipid interactions are known to soften gel networks and extend shelf-life by retarding starch re-crystallization [41,42].

Overall, the contrasting effects observed between DF and whole okara underscore their distinct molecular roles in starch gelation. DF primarily reinforces the gel matrix via its hydrophilic, water-binding, and network-stabilizing characteristics, while whole okara enhances cohesiveness and elasticity through combined protein–fiber–lipid interactions. This dual mechanism provides strategic formulation flexibility in high-fiber, protein-enriched starch-based food systems.

### 4.2. Crystalline Structure and Retrogradation Inhibition

The WAXS results provided insights into the structural transformations of rice starch (RS) in the presence of whole okara (RSO) and extracted dietary fiber (RSF), particularly regarding changes in crystallinity. Figure 6 shows the diffraction profiles of each sample group, while Figure 7 summarizes the crystallinity percentages derived from peak deconvolution.

#### 4.2.1. Diffraction Profile Changes and Crystallinity Trends

All samples retained diffraction characteristics corresponding to A-type polymorphs, with peak positions centered around 16–17° 2θ (Cu Kα). The A-type structure common to cereal starches, including rice starch, is defined by a monoclinic unit cell with a B2 symmetry group, as established in crystallographic studies [34,35]. This symmetry underlies the observed diffraction patterns and serves as the structural framework within which retrogradation-related changes occur.

Both RSF and RSO blends exhibited broader and less intense peaks than RS alone (Figure 6), indicating the disruption of long-range crystalline order. Crystallinity values (Figure 7) slightly increased with the addition of low-to-moderate levels of okara (RSO-5 and RSO-10) but decreased at higher substitution levels (RSO-15), suggesting alterations in molecular ordering within the monoclinic lattice rather than changes in polymorph type. In contrast, RSF treatments maintained or slightly enhanced crystallinity, with RSF-20 reaching 17.35%, higher than RS (16.48%). Notably, while peak broadening and intensity changes suggest a loss of structural coherence, they do not indicate a polymorphic transition, but rather reflect molecular-level disruptions within the A-type lattice.

#### 4.2.2. Influence of Fiber Composition and Molecular Interactions

Increased crystallinity in RSF mixtures may partly reflect the contribution of residual cellulose from dietary fiber. Cellulose I structures yield diffraction peaks in the same 2θ range as A-type starch (e.g., 15–17° and 22°), potentially inflating crystallinity estimates [26]. This phenomenon has been observed in potato-starch–okara systems, where dietary fiber addition did not alter crystal type but significantly increased total crystallinity. Thus, the diffraction intensity increase in RSF samples may not derive solely from retrograded starch, but may also from overlapping peaks of cellulose microfibrils [26,43].

Furthermore, the biochemical complexity of whole okara—containing ~29% protein and ~13% lipid—may impact starch retrogradation differently than purified DF. Lipids form amylose–lipid inclusion complexes (V-type) that hinder recrystallization ([41,42]). Proteins can also interact with starch via hydrogen bonding or steric effects. Yang et al. (2024) [38] reported that soybean protein isolate retarded rice starch retrogradation by limiting molecular mobility and chain alignment. These compositional factors may help to explain the irregular crystallinity response observed in RSO samples, reflecting less uniform molecular reordering.

#### 4.2.3. Mechanisms of Retrogradation Suppression by Dietary Fiber

The greater structural stability in RSF samples could result from several mechanisms. First, hydrogen bonding, as follows: Cellulose and hemicellulose have abundant hydroxyl groups that form hydrogen bonds with starch, interrupting starch–starch associations and limiting recrystallization. FTIR analyses in other studies confirmed these interactions [17]. Second, water competition and retention, as follows: Fibers sequester water, reducing its availability for starch chain reordering and thereby minimizing moisture loss and gel firming during storage [17,44]. Third, steric hindrance, as follows: Insoluble fiber particles interrupt the continuity of the starch matrix and physically impede chain alignment, further preventing retrogradation [17,45].

These effects, acting synergistically, explain the more pronounced retrogradation suppression in RSF samples compared to that observed in RSO samples. The higher purity and smaller particle size of RSF likely enhanced its interaction with the starch matrix, whereas the complex composition of RSO may have diluted these mechanisms. These findings are consistent with prior research, demonstrating that dietary fiber can mitigate starch retrogradation through both molecular-level interactions and physical structuring effects.

#### 4.2.4. Methodological Considerations and Future Perspectives

Despite meaningful trends in our data, limitations in WAXS analysis of starch–fiber systems must be acknowledged. The presence of overlapping peaks from cellulose, diffuse scattering patterns, and limited resolution in detecting amorphous contributions constrain the precision of crystallinity measurements. As Rodriguez-Garcia et al. (2021) [46] noted, resolving starch’s crystalline phases often requires improved indexing protocols and complementary structural techniques.

Future investigations may benefit from integrating synchrotron-based small-angle X-ray scattering (SAXS), Raman spectroscopy, or enzymatic deconvolution approaches to better differentiate fiber-derived diffraction from retrograded starch. These methods could help to clarify whether increases in crystallinity result from starch reordering or residual cellulose contributions.

In summary, WAXS findings indicate that whole okara and its extracted dietary fiber influence retrogradation through distinct mechanisms. RSF moderately increases crystallinity, likely due to hydrogen bonding and cellulose-related diffraction, while also limiting the development of extensive molecular order. In contrast, RSO disrupts crystalline structure more variably, reflecting the complex interactions of its protein, lipid, and fiber components. These insights advance our understanding of structure–function relationships in fiber-enriched starch systems and can inform the design of high-fiber, shelf-stable food products.

### 4.3. Molecular Interactions and Hydrogen Bonding

Fourier transform infrared (FTIR) spectroscopy provided valuable insights into the molecular interactions occurring within rice starch (RS) gels upon the incorporation of whole okara (RSO) and extracted dietary fiber (RSF). All fiber-containing samples exhibited increased O–H stretching band intensities (centered at 3280–3296 cm^−1^), indicating a denser hydrogen-bonding environment. This increase was more pronounced in RSF samples, suggesting that the purified, high-surface-area fiber interacted more effectively with starch hydroxyl groups, consistent with prior observations in dietary fiber–starch systems [17,36].

A new absorption band appeared consistently at ~1745 cm^−1^ in both RSO and RSF samples, corresponding to C=O stretching of ester carbonyl groups. This band was absent in native RS gels and is indicative of uronic acids or acetylated polysaccharides, likely derived from the fiber matrix [26,40]. Such carbonyl-associated bands are often used as spectral signatures for the presence of functionalized dietary fiber, such as hemicelluloses or fatty acid complexes, within starch-based matrices [10,40].

Changes in the fingerprint region (1200–900 cm^−1^), especially the relative intensities at 1047 and 1022 cm^−1^, provided further evidence of structural reorganization. The R_1047/1022_ ratio, widely used as an index of short-range molecular order or double-helical formation in retrograded starch [47,48], progressively increased with higher fiber addition. In this study, RSF-20 showed the highest ratio, followed closely by RSF-15 and RSO-20, indicating enhanced short-range ordering. These results align with findings by Wang et al. (2022) [16], who observed that extruded rice bran fiber increased hydrogen bonding and order in rice starch gels.

However, this ratio must be interpreted cautiously. While foundational FTIR work established its relevance to retrogradation [47], more recent investigations by Warren et al. (2016) [30] emphasized that the R_1047/1022_ ratio is sensitive to hydration, sample preparation, and baseline correction. From a vibrational perspective, the 1047 cm^−1^ band is associated with C–O stretching in more rigid, hydrogen-bonded double helices, while the 1022 cm^−1^ band corresponds to less constrained environments in amorphous regions. These assignments are supported by FTIR–XRD correlation studies and peak shift analyses in hydrated starch [30,49]. Moreover, the bands are composite in nature and may include overlapping vibrational modes from both starch and additive components. Thus, although a rising R_1047/1022_ ratio suggests enhanced molecular ordering, it is most robust as a relative metric rather than an absolute crystallinity measure. Moreover, given that FTIR was performed using an ATR accessory, we acknowledge that intensity is affected by sample-to-crystal contact. All samples were prepared and pressed under identical conditions, and spectra were normalized prior to ratio calculation. Therefore, the R_1047/1022_ ratio is interpreted as a relative trend metric, consistent across treatments.

These spectral shifts are supported by concurrent vibrational findings in related systems. Xia et al. (2014) [17] reported that cellulose and hemicellulose lowered the R_1047/1022_ ratio in retrograded rice starch, while causing a red-shift of the O–H stretch band, both indicative of stronger and more complex hydrogen bonding networks involving fiber–starch interactions. Similarly, in our RSF samples, the more intense O–H stretch and higher R_1047/1022_ ratios suggest that the purified fiber disrupted starch–starch associations while simultaneously promoting hetero-network formation between starch and fiber.

Protein and lipid components in okara may also contribute to these spectral features. Yang et al. (2024) [38] showed that soy protein isolate reduced retrogradation in rice starch by weakening short-range order, lowering the R_1047/1022_ ratio, and modifying the gel’s internal hydrogen bonding network. The presence of protein’s amide I/II bands and lipid-associated C=O peaks in FTIR spectra can signal such interactions [40]; however, in our study, the amide signals were masked or indistinct, likely due to low concentration or overlapping peaks.

In sum, the FTIR evidence supports a multi-faceted mechanism by which dietary fiber, and, to some extent, protein/lipid components in whole okara, modulate starch retrogradation. These mechanisms include hydrogen bonding competition, the incorporation of esterified or uronic acid groups, and steric interference within the gel network. When considered alongside WAXS-derived crystallinity and rheological properties, FTIR emerges as a sensitive tool for tracking local structural order and molecular-level interactions in starch–fiber systems.

### 4.4. Applications and Outlook

This study highlights the potential of okara-derived ingredients as functional modulators in starch-based systems. Extracted dietary fiber (DF), characterized by high insoluble fiber content (82.89%) and minimal residual protein or lipid, significantly enhanced gel stiffness, viscoelastic strength, and resistance to retrogradation. These properties make DF a suitable structural enhancer in applications requiring thermal and mechanical integrity—such as rehydratable noodle sheets, plant-based fillings, and gelled or layered desserts. Whole okara, by contrast, offered a more complex compositional matrix (38.21% total dietary fiber, 29.35% protein, and 12.85% fat), which imparted softer, more cohesive textures—suggesting its value in formulations such as fiber-enriched snacks, high-moisture meat analogues, or soft baked goods where pliability and nutritional enrichment are desirable.

The dual functionality of okara and DF—mechanical reinforcement vs. softening with protein/lipid enrichment—supports a design-based approach in food formulation, where the selection of fiber form is tailored to product needs. These findings also align with current interest in multifunctional fibers that not only contribute to health but also influence food texture, water mobility, and structural formation in complex systems.

Nevertheless, the findings must be contextualized within the limitations of this study. Experiments were conducted in simplified rice starch matrices under controlled lab conditions. The absence of competitive matrix components (e.g., sugars, salts, native proteins) and the absence of thermal or shear processing (beyond pre-gelatinization) may not fully reflect real-world complexity. While crystallinity data from WAXS showed informative trends, the presence of overlapping peaks and diffuse scattering limits the resolution of structural contributions from starch and fiber. These challenges raise questions about phase overlap and residual cellulose contributions, warranting further study using deconvolution techniques or SAXS.

Future research should advance beyond binary blends to explore how okara and DF perform under industrially relevant conditions, including high-temperature processing, pH variation, and long-term storage. Their synergy or antagonism with other structuring agents, such as hydrocolloids, emulsifiers, or plant proteins, also deserves attention, particularly in composite gels or extruded matrices. Furthermore, benchmarking against commercial soybean fiber isolates, like water-soluble polysaccharides or hemicellulose extracts, could establish clearer functionality comparisons and commercial pathways. Finally, sensory analysis and consumer validation are critical to ensuring that the physicochemical enhancements translate into acceptable and appealing food textures.

From a sustainability perspective, the valorization of okara into high-value fiber ingredients supports circular bioeconomy principles. By upgrading a nutrient-rich byproduct into functional texturizers, this work contributes to the growing demand for sustainable, plant-based food innovation.

## 5. Conclusions

This study demonstrates that whole okara and its extracted dietary fiber (DF) have distinct yet complementary effects on the retrogradation, gelation, and structural behavior of rice starch gels. DF, with its high insoluble fiber purity and minimal protein/lipid content, significantly reduced gelation temperature, reinforced gel elasticity, and more effectively suppressed crystallinity development during retrogradation compared to whole okara. These effects were supported by FTIR evidence of enhanced hydrogen bonding and the presence of ester carbonyl groups, particularly in DF-containing systems. In contrast, whole okara—which is rich in protein and lipids, alongside fiber—modulated gel properties by promoting cohesiveness and contributing to softer textures. These findings suggest that DF is well suited for structural reinforcement in starch-based formulations, while whole okara provides nutritional enrichment and textural modulation. Collectively, the results underscore the functional versatility of okara-derived ingredients and their value in advancing sustainable, fiber-enhanced food applications.

## Figures and Tables

**Table 1:** Dynamic rheological parameters of rice starch (RS), RS–okara (RSO), and RS–dietary-fiber (RSF) gels. Values are mean ± standard deviation (*n* = 3). Different superscript letters within each column indicate significant differences (*p* < 0.05) by one-way ANOVA and Tukey’s HSD test.

Sample	Tc (°C)	G′ at 0.01 rad/s (Pa) *	G′ at 1 rad/s (Pa)	G′ at 100 rad/s (Pa)	Frequency Dependence (Slope log G′ vs. log ω)	Structural Stability **
RS	75.3 ± 0.12 ^c^	4134 ± 50 ^a^	4480 ± 90 ^a^	4855 ± 146 ^a^	0.0175	Stable
RSO-5	66.8 ± 0.15 ^b^	4422 ± 88 ^b^	4783 ± 143 ^b^	5174 ± 103 ^b^	0.0171	Stable
RSO-10	66.7 ± 0.25 ^b^	4711 ± 85 ^c^	5087 ± 102 ^c^	5493 ± 121 ^c^	0.0167	Stable
RSO-15	66.6 ± 0.22 ^b^	4946 ± 99 ^d^	5341 ± 112 ^d^	5768 ± 115 ^d^	0.0167	Stable
RSO-20	66.7 ± 0.12 ^b^	5181 ± 145 ^e^	5595 ± 185 ^e^	6042 ± 181 ^e^	0.0167	Stable
RSF-5	59.5 ± 0.22 ^a^	14,307 ± 143 ^f^	17,590 ± 352 ^f^	21,627 ± 433 ^f^	0.0449	Stable
RSF-10	59.6 ± 0.13 ^a^	17,718 ± 354 ^g^	21,960 ± 549 ^g^	27,218 ± 299 ^g^	0.0466	Stable
RSF-15	59.7 ± 0.24 ^a^	21,130 ± 317 ^h^	26,330 ± 263 ^h^	32,809 ± 787 ^h^	0.0478	Stable
RSF-20	59.6 ± 0.12 ^a^	24,544 ± 491 ^i^	30,700 ± 675 ^i^	38,400 ± 307 ^i^	0.0486	Stable

* Storage modulus at critical low frequency (0.01 rad/s), indicating long-term gel stability [33]. ** Structural stability classification based on Mezger’s guideline (**G′** > 10 Pa at ω = 0.01 rad/s).

**Figure A1:**
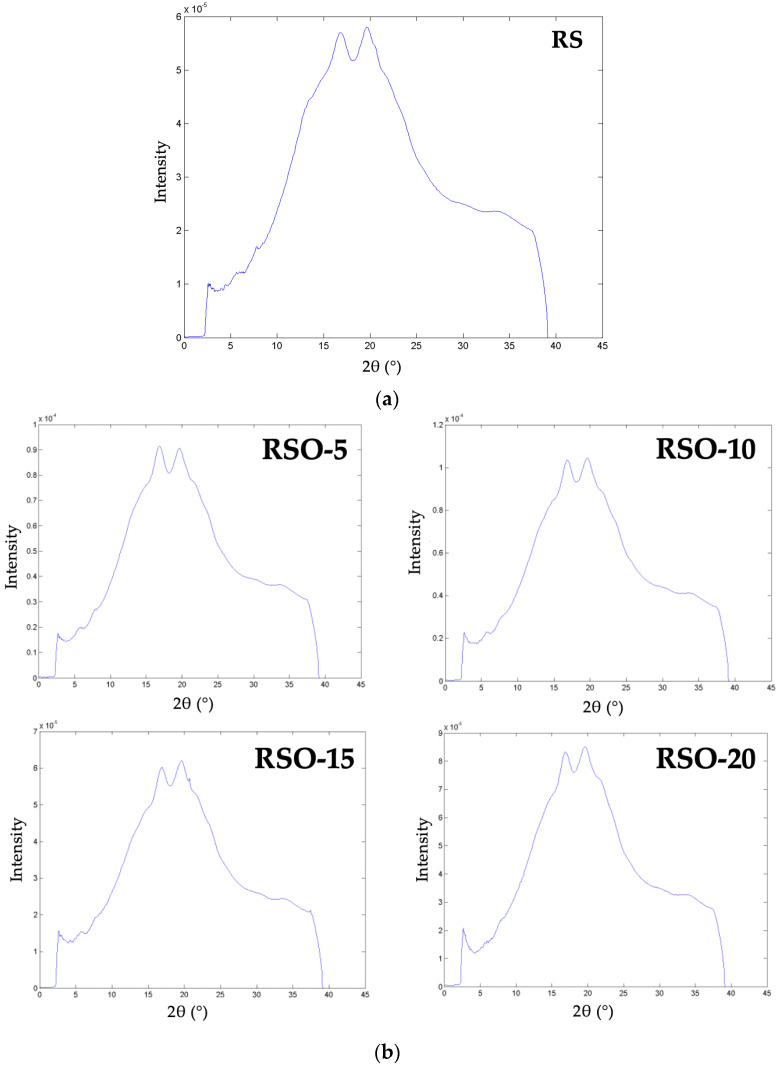

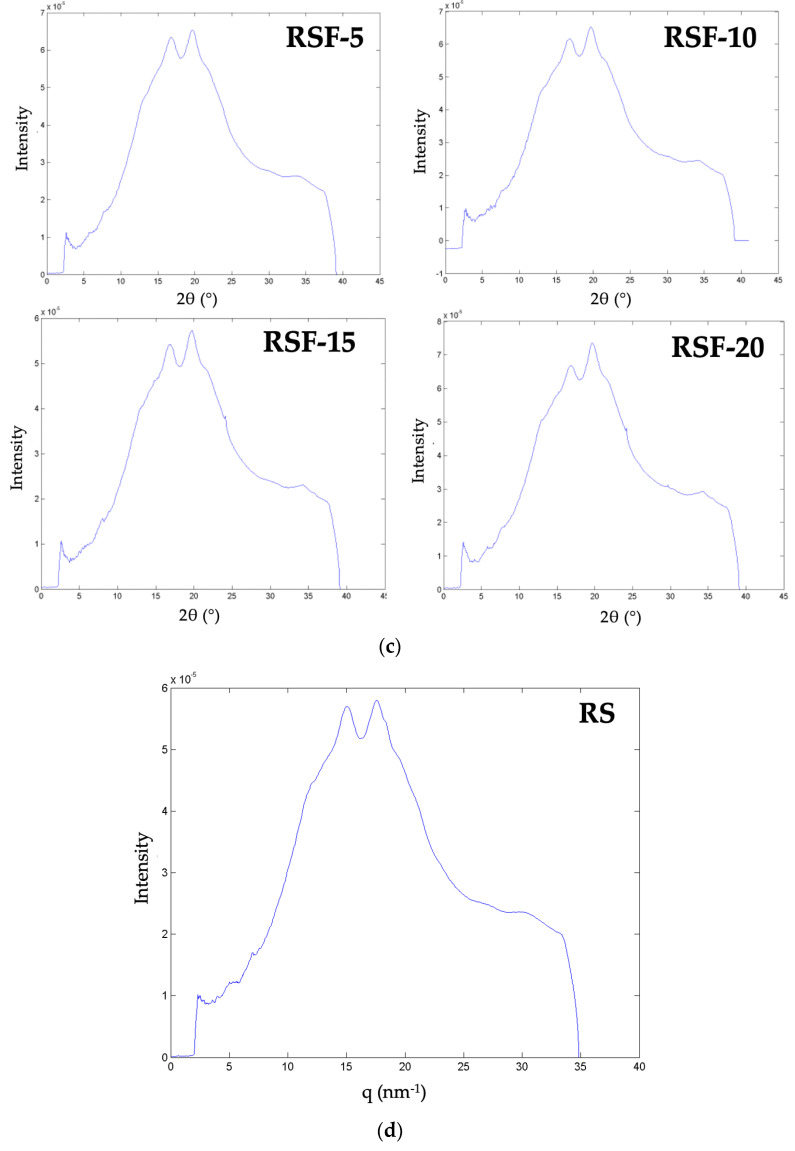

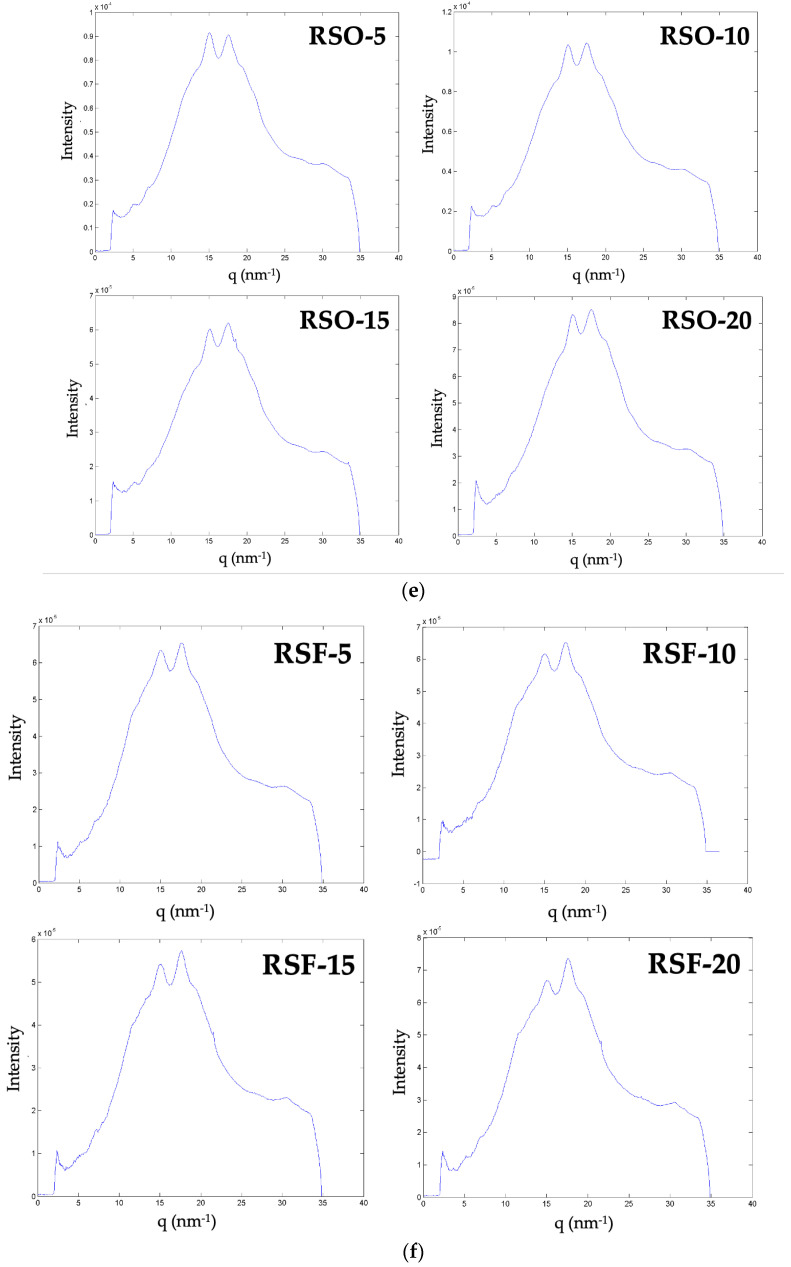
One-dimensional WAXS profiles of retrograded rice starch (RS) and its mixtures with whole okara (RSO) and extracted dietary fiber (RSF). Profiles are shown as follows: (**a**–**c**) plotted as intensity vs. scattering angle (2θ, Cu Kα); (**d**–**f**) plotted as intensity vs. scattering vector (q, nm⁻¹). Data were derived from synchrotron-based 2D WAXS images (λ = 1.3778 Å, 9 keV) and averaged over the full azimuthal range. All measurements were conducted at ambient temperature. The diffraction patterns show characteristic A-type monoclinic ordering in RS, with reduced peak intensity and broadening in fiber-treated samples, indicating disrupted long-range crystallite coherence.

## Data Availability

The original contributions presented in this study are included in the article, and further inquiries can be directed to the corresponding author.
